# Metabolic dysfunction‐associated liver disease predicts incident liver fibrosis in people with HIV mono‐infection: A cohort study

**DOI:** 10.1111/hiv.70079

**Published:** 2025-07-23

**Authors:** Juliana Fittipaldi, Sandra W. Cardoso, Estevão Portela Nunes, Cristiane Fonseca De Almeida, Patricia Dias De Brito, Valdilea G. Veloso, Beatriz Grinsztejn, Hugo Perazzo

**Affiliations:** ^1^ Evandro Chagas National Institute of Infectious Diseases (INI) Oswaldo Cruz Foundation (FIOCRUZ) Rio de Janeiro Brazil

**Keywords:** fibrosis, HIV, longitudinal studies, non‐alcoholic fatty liver disease

## Abstract

**Introduction:**

Metabolic dysfunction‐associated liver disease (MASLD) might progress to cirrhosis. We aimed to evaluate the association of MASLD with the risk of developing clinically significant fibrosis (CSF) in people living with HIV (PLWH).

**Methods:**

PLWH have been followed up since 2015 in the PROSPEC‐HIV cohort (NCT02542020) with questionnaires, blood samples, and transient elastography (TE) on the same day. Participants with viral hepatitis, who were c‐ART naïve, those with CSF at baseline or unreliable TE examinations and who lost follow‐up were excluded. Liver steatosis and fibrosis were assessed by TE‐Controlled Attenuation Parameter (CAP) and TE‐liver stiffness measurement (LSM), respectively. MASLD was defined as the presence of steatosis (CAP ≥263 dB/m) at baseline with at least one cardiometabolic risk factor without hazardous alcohol intake [Alcohol Use Disorders Identification Test (AUDIT) score <8]. The primary outcome was the development of CSF (LSM ≥8.0 kPa) during follow‐up. Kaplan–Meier curves and Cox proportional hazards multivariate models were performed.

**Results:**

A total of 304 participants with HIV mono‐infection under c‐ART and without liver fibrosis (43.4% male, median [interquartile range, IQR] age = 44 [IQR, 36–52] and body mass index [BMI] = 25.6 [23.0–29.0] kg/m^2^ and 17.8% with MASLD) were included. During a median follow‐up of 7.4 (IQR, 6.0–8.3) years, 11.8% (*n* = 36) participants developed CSF. The cumulative incidence of CSF at the 8th year was higher in PLWH with MASLD than in those without (30.1% [95% CI, 18.0–47.6] vs. 8.8% [95% CI, 5.4–14.2], *p* < 0.001). MASLD at baseline was significantly associated with the incidence of CSF in a multivariate Cox model (adjusted hazard ratio [aHR] = 2.92 [95% CI, 1.40–6.09]).

**Conclusion:**

MASLD increased the risk of liver fibrosis in people with HIV mono‐infection under c‐ART.

## INTRODUCTION

Metabolic dysfunction‐associated liver disease (MASLD), formerly known as non‐alcoholic fatty liver disease (NAFLD), is characterized by hepatic steatosis associated with at least one cardiometabolic risk factor, without hazardous alcohol intake [1, 2]. MASLD is a major public health issue in the general population [3]. The natural history of this liver disease might range from simple steatosis to severe clinical presentations, such as steatohepatitis (NASH/MASH) and advanced fibrosis/cirrhosis [4].

In the last decades, the life expectancy of people living with HIV (PLWH) has improved due to a high coverage of combined antiretroviral therapy (c‐ART) [5]. Therefore, PLWH have been more exposed to noncommunicable diseases (NCDs), including metabolic diseases [6]. PLWH with liver steatosis were at high risk of cardiovascular disease in a multi‐region analysis including 1750 participants from 9 low‐to‐middle income countries [7]. Since PLWH have been more exposed to NCDs, such as obesity and type 2 diabetes, several studies have reported the burden of MASLD in this population [8, 9, 10]. A recent systematic review and meta‐analysis has reported a pooled prevalence of MASLD of 34% (95% CI 30–38) among PLWH [11]. However, the burden of this liver disease in PLWH remains extremely variable across countries/regions, since the prevalence of MASLD ranged from 20% in sub‐Saharan Africa to around 40% in Central/Latin America [12].

A long‐term longitudinal study has shown that fibrosis stages rather than the presence of steatohepatitis predict overall mortality in the general population [13]. PLWH and MASLD seem to have a higher prevalence of liver‐biopsy‐proven fibrosis than matched uninfected MASLD controls [14]. Few studies have assessed long‐term fibrosis progression. Since PLWH are at high risk of MASLD and fibrosis stage remains strongly associated with liver‐specific outcomes, it is critical to identify the relationship between MASLD and the incidence of liver fibrosis in PLWH, especially in limited‐resource settings. Therefore, we aimed to evaluate the association of MASLD with the long‐term risk of developing clinically significant/advanced fibrosis in people with HIV mono‐infection in Rio de Janeiro, Brazil.

## METHODS

### PROSPEC‐HIV study

The PROSPEC‐HIV (NCT02542020) study is a prospective cohort that has been following a convenience sample of 744 outpatients aged ≥18 years living with HIV infection at INI/FIOCRUZ (Rio de Janeiro, Brazil) since July 2015. Participants from the PROSPEC‐HIV study have been submitted to anthropometric measures, questionnaires, fasting blood samples, and transient elastography (TE) by Fibroscan (EchoSens, Paris, France) on the same day every 36 months. [15] TE has been assessing liver fibrosis by liver stiffness measurement (LSM) and hepatic steatosis by Controlled Attenuation Parameter (CAP). The protocol visits were postponed from March 2020 to June 2023 due to the COVID‐19 pandemic in Brazil. The study protocol was approved by the Ethics Committee from INI/FIOCRUZ (IRB 32889514.4.0000.5262). All participants signed an informed consent form prior to enrolment in the PROSPEC‐HIV study. Clinical Trial Registration: NCT02542020.

### Study design and population

All 744 participants included in the PROSPEC‐HIV study were eligible for this longitudinal analysis. The exclusion criteria were the presence of viral hepatitis (positive HBsAg and/or detectable HCV‐RNA) or a history of previous HCV sustained virological response (HCV‐SVR), c‐ART naïve or the absence of CAP measure at the baseline visit, unreliable LSM or incident viral hepatitis at any visit and loss to follow‐up (defined by the absence of at least two TE examinations to assess liver fibrosis progression). Additionally, participants with clinically significant fibrosis (CSF, defined as LSM ≥8 kPa) were excluded. Therefore, this study included people with HIV mono‐infection under c‐ART without liver fibrosis at baseline who were prospectively followed up in the PROSPEC‐HIV study.

### Data collection and blood sample

The PROSPEC‐HIV study included the following data collection at every visit: (i) anthropometric measures (weight, height, waist and hip circumference); (ii) blood pressure; (iii) history of co‐morbidities, use of medications and physical activity (minutes of moderate aerobic activity per week) and (iv) alcohol intake (Alcohol Use Disorders Identification Test [AUDIT score]) and smoking (never, past or current) (Data S1). Overweight and obesity were defined as body mass index (BMI) ≥25 and ≥30 kg/m^2^, respectively. Additionally, the PROSPEC‐HIV study included the following blood analyses at every visit: fasting glucose, liver tests (alanine transferase [ALT], aspartate transferase [AST] and gamma‐glutamyl‐transferase [GGT]), total bilirubin, albumin, lipid profile (total cholesterol, high‐density lipoprotein [HDL] and low‐density lipoprotein [LDL] cholesterol, as well as triglycerides, mg/dL) and platelet count. All blood tests were performed in a centralized laboratory with a Dimension‐RxL‐Max analyser (Siemens Healthcare Diagnostic, Deerfield, IL, USA).

### Transient elastography

TE by Fibroscan was performed in every visit of the PROSPEC‐HIV study by an experienced operator (HP or JF) following a validated procedure after an overnight fasting (17). CAP and LSM results were considered reliable when all the following criteria had been met: (i) 10 successful measurements; (ii) an interquartile range (IQR) lower than 30% of the median value of LSM for fibrosis and CAP for steatosis; and (iii) a success rate of more than 60% [16].

### HIV infection and c‐ART history

INI/FIOCRUZ has been maintaining an electronic longitudinal clinical database of PLWH since 1990 (INI/FIOCRUZ HIV cohort). This database has been regularly updated by trained investigators using medical records, laboratory results and pharmacy records, including c‐ART prescription [17]. The following data were collected at the INI/FIOCRUZ HIV cohort: (i) duration of HIV infection (based on the date of the first positive HIV antibody test); (ii) information on c‐ART regimens (medications names, initiation and discontinuation dates); (iii) duration of antiretroviral therapy (based on the date of the first initiation of any antiretroviral drug); and (iv) CD4+ T lymphocyte count, HIV viral load and viral hepatitis serologies. Results from the most recent CD4+ T lymphocyte count and HIV‐1 RNA test obtained prior to or after the baseline visit were used in the analysis. Undetectable HIV viral load was defined as HIV‐1 RNA ≤100 copies/mL.

### Metabolic dysfunction‐associated liver disease (MASLD)

The presence of steatotic liver disease (SLD) was defined as the presence of hepatic steatosis (CAP ≥263 dB/m). Cardiometabolic risk factors were defined as follows: (i) overweight/obesity: BMI ≥25 kg/m^2^ or waist circumference ≥102 cm in males or 88 cm in females; (ii) pre/type 2 diabetes: serum fasting glucose ≥100 mg/dL or treatment; (iii) hypertension: blood pressure ≥130/85 mmHg or specific drug treatment; (iv) hypertriglyceridaemia: plasma triglycerides ≥150 mg/dL or triglycerides‐lowering treatment (such as fibrates); and (v) low‐HDL‐c: plasma HDL‐cholesterol ≤40 mg/dL for males and ≤50 mg/dL for females or lipid‐lowering agents [1]. Hazardous alcohol intake was defined as AUDIT score ≥8. MASLD was defined as SLD in the presence of at least one cardiometabolic risk factor without hazardous alcohol consumption (AUDIT <8). Metabolic dysfunction‐ and alcohol‐associated liver disease (Met‐ALD) was defined as the presence of SLD, at least one metabolic dysfunction‐associated feature, and hazardous alcohol intake defined as AUDIT from 8 to 15. Additionally, alcohol‐related liver disease (ALD) was determined by excessive alcohol intake (AUDIT >15) [1, 18].

### Outcomes

The primary outcome of this analysis was to assess the incidence of CSF (LSM ≥8.0 kPa) during follow‐up through the PROSPEC‐HIV study in people with HIV mono‐infection under c‐ART and without liver fibrosis at baseline. The first TE examination performed in the PROSPEC‐HIV visits with CSF during follow‐up was considered for the analysis. All TE examinations performed during the PROSPEC‐HIV study were considered. Participants without CSF during follow‐up were censored at the last visit up to 31 October 2024. A sensitivity analysis was performed to identify risk factors associated with advanced fibrosis (LSM ≥9.5 kPa).

### Statistical analysis

Categorical variables were described as absolute numbers (*n*) and relative frequencies (%), while continuous variables were reported as median and IQR. For comparing independent groups, either the chi‐squared (proportions) or Mann–Whitney test (quantitative variables) was used. The duration of follow‐up was calculated from baseline to the date of the primary outcome (LSM ≥8.0 kPa) or last visit within the PROSPEC‐HIV study up to the censored date (31 October 2024) for those with LSM <8.0 kPa during follow‐up. The incidence rates of the primary outcome (per 1000 person‐years [PY]) and the relative risk (RR) of incidence of the outcome according to socio‐demographic and clinical features, laboratory results, classes of c‐ART and presence/absence of MASLD were assessed. Kaplan–Meier curves were plotted (log‐rank *p* value). Cox proportional hazards models were performed. Variables found to be significantly associated (*p* ≤ 0.05) with the incidence of CSF were entered into the multivariate Cox models adjusted for the following confounding factors for liver fibrosis progression: age (per 10 years), sex at birth (male vs. female), physical activity (≥150 min vs. <150 min per week), alcohol intake (AUDIT ≥8 vs. <8) and CD4 count (<350 vs. ≥350 cells/mm^3^). If associated with the outcome, MASLD was entered into the multivariate Cox Model A and replaced by metabolic features in the multivariate Cox Model B to avoid collinearity. The software STATA 18.0 SE‐Standard Edition for Windows (StataCorp LP, College Station, TX, USA) was used for statistical analysis.

## RESULTS

### Study population

**FIGURE 1:**
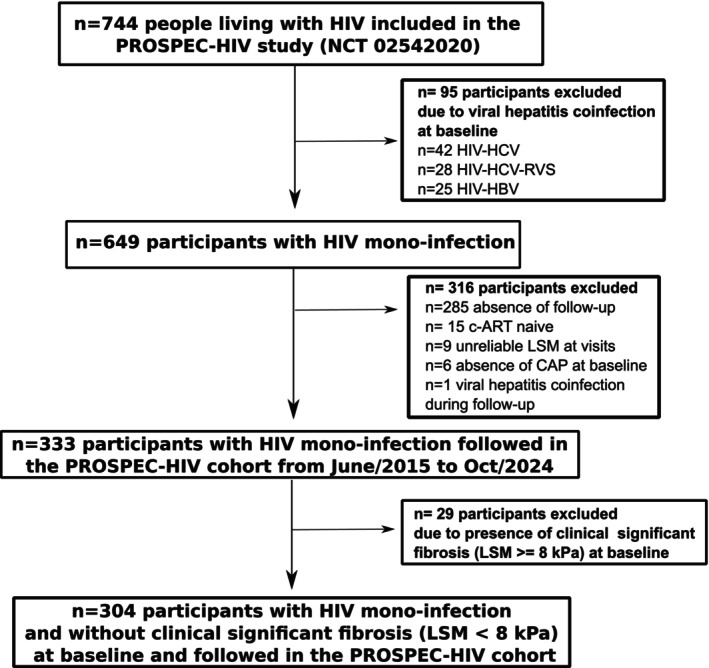
Flow chart of the study.

A total of 744 participants were recruited for the PROSPEC‐HIV study from June 2015 to October 2019. Of them, 95 subjects were excluded due to the presence of viral hepatitis co‐infection (HBV‐HIV, *n* = 25; HCV‐HIV, *n* = 42) or previous HCV cure (HCV‐SVR, *n* = 28) at baseline. Of the 649 participants with HIV mono‐infection, 316 individuals were excluded due to the absence of follow‐up (*n* = 285), c‐ART naïve (*n* = 15), unreliable LSM at visits (*n* = 9), missing CAP measure at baseline (*n* = 6) and new HCV infection during follow‐up (*n* = 1). Of the 333 participants with HIV mono‐infection followed up with LSM and CAP by TE from June 2015 to October 2024 through the PROSPEC‐HIV cohort, 29 subjects (8.7%) were excluded due to the presence of CSF at baseline (Figure 1). All participants included in this analysis had at least two reliable LSM for fibrosis assessment. Therefore, 304 patients with HIV mono‐infection under c‐ART therapy and without liver fibrosis at baseline (43.4% male, median age = 44 [IQR, 36–52], 55% with overweight/obesity and 26.6% with type 2 diabetes) were included in this analysis. Regarding HIV history, the median (IQR) duration of HIV infection was 9.6 (5.3–15.7) years, and participants were under c‐ART for a median time of 7.1 (3.7–13.4) years. The median CD4 + T lymphocyte count was 671 (IQR, 471–871) cells/mm^3^; 88.5% of participants had undetectable HIV‐RNA, 50% of individuals were currently using protease inhibitor (PI) and 6.9% were under integrase strand transfer inhibitors (INSTI) at baseline. Participants were under PI for 1.56 years (IQR, 0.0–7.6) years. Additionally, subjects had a median cumulative use of AZT, ddI, d4T or ddC drugs of 1.3 (IQR, 0.0–8.2) years. The prevalence (95% CI) of MASLD (*n* = 54), Met‐ALD (*n* = 14) and ALD (*n* = 2) at baseline were 17.8% (13.9–22.5), 4.6% (2.7–7.6) and 0.6% (0.2–2.6), respectively. All participants with MASLD would also be classified as NAFLD. Table 1 describes the characteristics of participants at baseline.

**TABLE 1:** Socio‐demographic, clinical and laboratory characteristics of participants at baseline (*n* = 304).

	All (*n* = 304)
Demographic characteristics	
Male sex at birth^a^	132 (43.4)
Age, years^b^	44 (36–52)
Skin colour^a^	
White	123 (40.5)
Black	65 (21.4)
Brown	114 (37.5)
Other/unknown	2 (0.6)
Metabolic factors	
BMI, kg/m^2b^	25.6 (23.0–29.0)
BMI categories^a^	
BMI <25.0 kg/m^2^	137 (45.1)
BMI ≥25.0–29.9 kg/m^2^	109 (35.9)
BMI ≥30 kg/m^2^	58 (19.1)
Type‐2 diabetes^a^	81 (26.6)
Hypertension^a^	102 (33.6)
Dyslipidaemia^a^	174 (57.2)
Lifestyle	
Physical activity >150 min/week^a^	75 (24.6)
Smoking^a^	
Never	185 (60.9)
Former	66 (21.7)
Smoker	53 (17.4)
Alcohol intake	
AUDIT score^b^	3 (0–8)
AUDIT score ≥8^a^	76 (25.0)
Biochemistry	
ALT, UI/L^b^	29 (23–41)
AST, UI/L^b^	25 (20–33)
GGT, U/L^b^	44 (32–71)
Alkaline phosphatase, U/L^b^	89 (72–110)
Albumin, g/dL^b^	3.9 (3.7–4.2)
Fasting glucose, mg/dL^b^	92 (88–99)
Total cholesterol, mg/dL^b^	186 (160–219)
HDL‐cholesterol, mg/dL^b^	43 (37–54)
LDL‐cholesterol, mg/dL^b^	114.5 (92–140)
Triglycerides, mg/dL^b^	121 (84–168)
Platelet count, ×10^9^/mm^3b^	250 (211–290)
HIV infection and c‐ART history	
Duration of HIV infection, years^b^	9.6 (5.3–15.7)
CD4 count, cells/mm^3b^	671 (471–871)
CD4 categories^a^	
CD4 count ≥500 cells/mm^3^	216 (71.1)
CD4 count 499–350 cells/mm^3^	40 (13.2)
CD4 count <350 cells/mm^3^	40 (13.2)
Undetectable HIV‐RNA (<100 copies/mL)^a^	269 (88.5)
Duration of c‐ART, years^b^	7.1 (3.7–13.4)
Current core‐ART drugs^a^	
Protease inhibitor	152 (50.0)
Integrase strand transfer inhibitor	21 (6.9)
Transient elastography	
Probe M/XL^a^	291 (95.7)/13 (4.3)
Liver stiffness measurement (LSM)	
LSM, kPa^b^	5.2 (4.4–6.2)
IQR/LSM ratio, %^b^	12 (9–17)
Controlled Attenuation Parameter (CAP)	
CAP, dB/m^b^	227 (196–262)
IQR of CAP, dB/m^b^	32 (23.5–44.5)
Steatotic liver disease (SLD)^a^	
No SLD	234 (77.0)
MASLD	54 (17.8)
Met‐ALD	14 (4.6)
ALD	2 (0.6)

*Note*: Data expressed as (a) absolute (%) or (b) median (IQR). Presence of steatosis was defined by CAP ≥263 dB/m. MASLD was defined as presence of steatosis with at least one cardiometabolic risk factor without excessive alcohol intake. Physical activity was assessed in minutes of moderate‐intensity physical activity per week. Missing data (*n*): age (*n* = 4), type 2 diabetes (*n* = 2), hypertension (*n* = 6), current core‐ART (20); dyslipidaemia (*n* = 9), ALT (*n* = 1), AST (*n* = 1), GGT (*n* = 1), alkaline phosphatases (*n* = 2), albumin (*n* = 2), fasting glucose (*n* = 1), total cholesterol (*n* = 3), HDL‐c (*n* = 17), LDL‐c (*n* = 18), triglycerides (*n* = 4), platelet count (*n* = 1), HIV‐RNA (*n* = 1) and CD4 count (*n* = 8).

Abbreviations: ALD, alcohol liver disease; ALT, alanine transferase; AST, aspartate transferase; AUDIT, Alcohol Use Disorders Identification Test; BMI, body mass index; CAP, Controlled Attenuation Parameter; c‐ART, combined antiretroviral therapy; HDL, high‐density lipoprotein; LDL, low‐density lipoprotein; LSM, liver stiffness measurement; MASLD, metabolic dysfunction‐associated steatotic liver disease; Met‐ALD, metabolic dysfunction‐ and alcohol‐associated liver disease; PI, protease inhibitor; SLD, steatotic liver disease.

### Incidence of CSF

A total of 36 (11.8%) participants developed CSF during a median follow‐up of 7.4 (IQR, 6.0–8.3) years. People with incident CSF had a higher proportion of participants with obesity (52.8% vs. 14.6%, *p* < 0.001), a higher proportion of hypertension (66.7% vs. 29.1%, *p* < 0.001) and higher ALT and AST levels compared to those who did not develop CSF. Additionally, the prevalence of MASLD (47.2% vs. 13.8%) and Met‐ALD (8.3% vs. 4.9%) was significantly higher among participants with incident CSF (*p* < 0.001) than among those without (Table 2). The characteristics regarding the HIV infection were similar among people with or without incident CSF.

**TABLE 2:** Socio‐demographic, clinical and laboratory characteristics of participants at baseline according to developing clinically significant fibrosis (CSF) during follow‐up.

	PLWH without incident CSF (*n* = 268)	PLWH with incident CSF (*n* = 36)	*p* value
Demographic characteristics			
Male sex at birth^a^	114 (42.5)	18 (50.0)	0.400
Age, years^b^	44 (36–52)	49 (40–55)	0.085
Black/Brown skin colour^a^	158 (59.0)	21 (58.3)	0.920
Metabolic factors			
Obesity, BMI >30 kg/m^2a^	39 (14.6)	19 (52.8)	<0.001
Type 2 diabetes^a^	67 (25.0)	14 (38.9)	0.061
Hypertension^a^	78 (29.1)	24 (66.7)	<0.001
Dyslipidaemia^a^	150 (56.0)	24 (66.7)	0.140
Lifestyle			
Physical activity >150 min/week^a^	67 (25.0)	8 (22.2)	0.720
Smoker^a^	47 (17.5)	6 (16.7)	0.900
Hazard alcohol intake, AUDIT score ≥8^a^	68 (25.4)	8 (22.2)	0.680
Biochemistry			
ALT, UI/L^b^	28 (22–40)	34 (25–50)	0.030
AST, UI/L ^b^	25 (20–32)	28 (24–38)	0.006
GGT, U/L^b^	43 (31–69)	58 (37–87)	0.004
Alkaline phosphatase, U/L^b^	89 (72–110)	89 (65–113)	0.93
Albumin, g/dL^b^	3.9 (3.7–4.1)	4.0 (3.7–4.3)	0.27
Fasting glucose, mg/dL^b^	92 (88–99)	99 (88–105)	0.033
Total cholesterol, mg/dL^b^	185 (158–219)	198 (169–225)	0.20
HDL‐cholesterol, mg/dL^b^	42 (36–54)	45 (37–52)	0.50
LDL‐cholesterol, mg/dL^b^	113 (91–139)	123 (101–148)	0.22
Triglycerides, mg/dL^b^	121 (82–165)	120 (94–172)	0.52
Platelet count, ×10^9^/mm^3b^	252 (214–290)	238 (208–290)	0.46
HIV infection and c‐ART history			
Duration of HIV infection, years^b^	9.2 (5.2–15.3)	12.7 (6.3–17.9)	0.19
CD4 count <350 cells/mm^3b^	667 (470–861)	697 (571–965)	0.31
Undetectable HIV‐RNA (<100 copies/mL)^a^	32 (11.9)	2 (5.6)	0.250
Duration of c‐ART, years^b^	7.0 (3.8–13.5)	8.1 (2.3–13.4)	0.80
Current use of protease inhibitor	137 (51.1)	15 (41.7)	0.240
Current use of INSTI	19 (7.1)	3 (8.3)	0.800
Liver disease			<0.001
No SLD	218 (81.3)	16 (44.4)	
MASLD	37 (13.8)	17 (47.2)	
Met‐ALD	11 (4.1)	3 (8.3)	
ALD	2 (0.8)	0 (0.0)	

*Note*: Data expressed as (a) absolute (%) or (b) median (IQR). MASLD was defined as presence of steatosis with at least one cardiometabolic risk factor without excessive alcohol intake. Physical activity was assessed in minutes of moderate‐intensity physical activity per week. Missing data (*n*): age (*n* = 4), type 2 diabetes (*n* = 2), hypertension (*n* = 6), dyslipidaemia (*n* = 9), ALT (*n* = 1), AST (*n* = 1), GGT (*n* = 1), alkaline phosphatases (*n* = 2), albumin (*n* = 2), fasting glucose (*n* = 1), total cholesterol (*n* = 3), HDL‐c (*n* = 17), LDL‐c (*n* = 18), triglycerides (*n* = 4), platelet count (*n* = 1), HIV‐RNA (*n* = 1) and CD4 count (*n* = 8).

Abbreviations: ALD, alcohol liver disease; ALT, alanine transferase; AST, aspartate transferase; AUDIT, Alcohol Use Disorders Identification Test; BMI, body mass index; c‐ART, combined antiretroviral therapy; CSF, clinically significant fibrosis (defined by LSM ≥8 kPa during follow‐up); HDL, high‐density lipoprotein; INSTI, integrase strand transfer inhibitors; LDL, low‐density lipoprotein; MASLD, metabolic dysfunction‐associated steatotic liver disease; Met‐ALD, metabolic dysfunction‐ and alcohol‐associated liver disease; SLD, steatotic liver disease.

**FIGURE 2:**
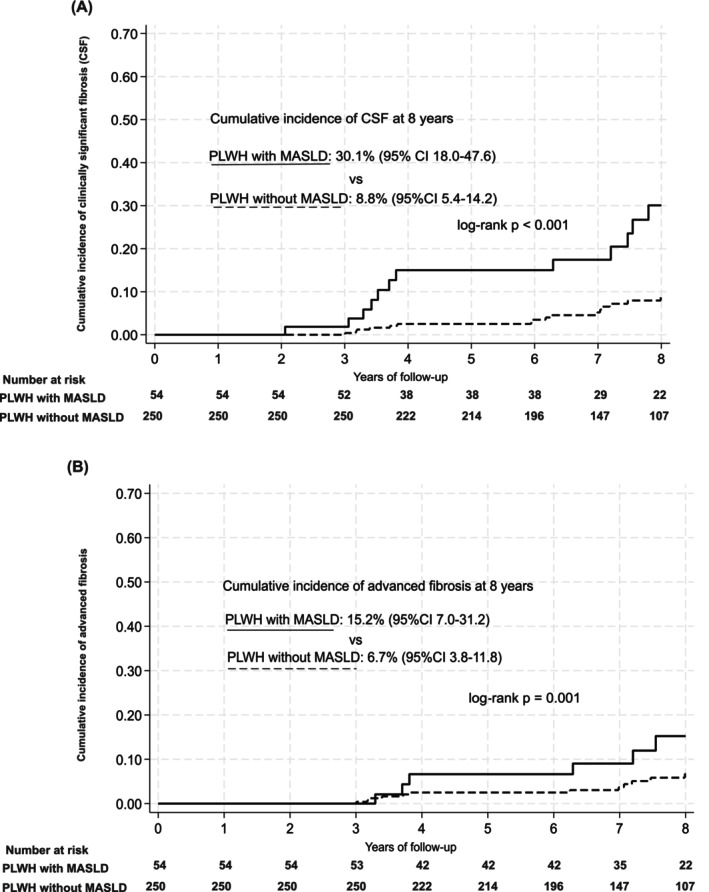
Cumulative incidence of (a) clinically significant fibrosis (CSF) defined as liver stiffness measurement (LSM) ≥8 kPa and (b) advanced fibrosis defined as LSM ≥9.5 kPa according to the presence or absence of metabolic dysfunction‐associated liver disease (MASLD) in people with HIV mono‐infection.

The overall incidence rate of CSF was 17.3 (12.5–23.9) per 1.000 PY. The RR (95% CI) of developing CSF during follow‐up was significantly higher in people with obesity (5.03 [2.62–9.68], *p* < 0.001), type 2 diabetes (2.60 [1.32–5.11], *p* = 0.004), hypertension (4.02 [2.01–8.03], *p* < 0.001) and MASLD (4.55 [2.37–8.76], *p* < 0.001) than in those without (Table 3). The cumulative incidence (% [95% CI]) of CSF was significantly higher in people with MASLD than in those without [30.1% (18.0–47.6) vs. 8.8% (5.4–14.2); log‐rank *p* < 0.001] (Figure 2a). Among the participants without MASLD (*n* = 250), a total of 7.6% (*n* = 19) developed CSF during the follow‐up [incidence rate = 10.9 (95% CI, 7.0–17.1) per 1.000 PY]. In a sensitivity analysis, the overall incidence rate of advanced liver fibrosis during follow‐up was 12.3 (95% CI 8.4–18.1) per 1.000 PY (*n* = 26 outcomes) (Table S1). Similarly, the cumulative incidence [% (95% CI)] of advanced fibrosis was significantly higher in people with MASLD than in those without [15.2% (7.0–31.2) vs. 6.7% (3.8–11.8); log‐rank *p* < 0.001] (Figure 2b).

**TABLE 3:** Incidence rate (per 1000 person‐years [95% confidence interval]) of clinically significant fibrosis (CSF; LSM ≥8 kPa) in people living with HIV mono‐infection without liver fibrosis (LSM <8 kPa) at baseline followed up in the PROSPEC‐HIV cohort during a median time of 7.4 (IQR, 6.0–8.3) years.

	*n*	Number of outcomes	Incidence of CSF per 1000 PY (95% CI)	Relative risk (95% CI)	*p* value
Overall	304	36	17.3 (12.5–23.9)		
According to sex at birth					
Female	172	18	15.1 (9.50–23.9)	Reference	
Male	132	18	20.2 (12.7–32.0)	1.34 (0.70–2.57)	0.380
According to age					
Age <50 years	208	20	13.9 (8.9–21.5)	Reference	
Age ≥50 years	92	16	26.0 (15.9–42.4)	1.88 (0.97–3.62)	0.057
According to metabolic features					
Absence of obesity (BMI <30 kg/m^2^)	246	17	10.0 (6.2–16.0)	Reference	
Presence of obesity (BMI ≥30 kg/m^2^)	58	19	50.1 (32.0–78.6)	5.03 (2.62–9.68)	<0.001
Absence of type 2 diabetes	221	21	12.7 (8.3–19.5)	Reference	
Presence of type 2 diabetes	81	14	33.1 (19.6–55.8)	2.60 (1.32–5.11)	0.004
Absence of hypertension	196	12	8.8 (5.0–15.4)	Reference	
Presence of hypertension	102	14	35.2 (23.6–52.6)	4.02 (2.01–8.03)	<0.001
Absence of dyslipidaemia	121	10	11.8 (6.3–21.9)	Reference	
Presence of dyslipidaemia	174	24	20.4 (13.7–30.5)	1.74 (0.83–3.63)	0.138
According to ALT levels					
Normal ALT levels (<40 U/L)	222	21	13.8 (9.0–21.2)	Reference	
Abnormal ALT levels (≥40 U/L)	81	14	24.9 (14.8–42.1)	1.80 (0.92–3.54)	0.083
According to HIV control					
CD4 count ≥350 cells/mm^3^	256	30	17.1 (11.9–24.4)	Reference	
CD4 count <350 cells/mm^3^	40	5	17.9 (7.4–42.9)	1.05 (0.41–2.70)	0.925
Undetectable HIV viral load	269	34	18.4 (13.1–25.7)	Reference	
Detectable HIV viral load	34	2	8.8 (2.2–35.2)	0.48 (0.12–1.99)	0.301
According to use of INSTI					
No use of INSTI during follow‐up	161	20	18.4 (11.8–28.5)	Reference	
Use or switch to INSTI during follow‐up	143	16	16.1 (9.8–26.2)	0.87 (0.45–1.69)	0.688
Cumulative use of INSTI <12 months	164	18	16.4 (10.3–26.0)	Reference	
Cumulative use of INSTI ≥12 months	140	18	18.2 (11.5–28.9)	1.11 (0.58–2.14)	0.747
According to liver disease					
Absence of MASLD	250	19	10.9 (7.0–17.1)	Reference	
Presence of MASLD	54	17	49.6 (30.8–79.8)	4.55 (2.37–8.76)	<0.001

*Note*: Missing data for age (*n* = 300 and outcomes = 36); type‐2 diabetes (*n* = 302 and outcomes = 35); hypertension (*n* = 298 and outcomes = 36); dyslipidaemia (*n* = 295 and outcomes = 34); ALT levels (*n* = 303 and outcomes = 35); CD4 count (*n* = 296 and outcomes = 35); HIV viral load (*n* = 303 and outcomes = 36).

Abbreviations: INSTI, integrase strand transfer inhibitors; LSM, liver stiffness measurement; MASLD, metabolic dysfunction‐associated steatotic liver disease.

### Factors associated with incident CSF

The presence of MASLD [adjusted hazard ratio (aHR) = 4.80 (95% CI 2.20–10.5)] was significantly associated with increased risk of developing CSF (LSM >8 kPa) during follow‐up in a Cox proportional multivariate model (Model A—Table 4). In multivariate Cox Model B [aHR (95% CI)], when replacing MASLD by the individual cardiometabolic risk factors, obesity [BMI ≥30 kg/m^2^, aHR = 4.28 (1.96–9.33)] and hypertension [aHR = 3.18 (1.30–7.76)] were independently associated with the incidence of CSF. Type 2 diabetes was associated with the risk of incident CSF in the univariate analysis [hazard ratio (HR) = 3.42 (95% CI 1.73–6.78)], but not after adjustment for confounding factors [aHR = 1.56 (95% CI 0.70–3.48)] (Table 4). Similarly, obesity [HR = 3.42 (95% CI, 1.61–7.27), *p* = 0.001] and hypertension [HR = 3.49 (95% CI, 1.49–8.16), *p* = 0.004] were independently associated with the incidence of CSF in people without MASLD (*n* = 250) (Table S2).

**TABLE 4:** Cox proportional hazards models to identify risk factors associated with incidence of clinically significant fibrosis (*n* = 36; LSM ≥8 kPa) in people living with HIV mono‐infection without liver fibrosis (LSM <8 kPa) at baseline followed up in the PROSPEC‐HIV cohort (*n* = 304) during a median time of 7.4 (IQR, 6.0–8.3) years.

	Univariate analysis		Multivariate Model A		Multivariate Model B	
	HR (95% CI)	*p* value	aHR (95% CI)	*p* value	aHR (95% CI)	*p* value
Socio‐demographic characteristics and lifestyle						
Male sex at birth	1.43 (0.74–2.75)	0.282	1.36 (0.69–2.66)	0.376	1.21 (0.56–2.63)	0.632
Age (per 10 years)	1.32 (0.97–1.79)	0.075	1.21 (0.86–1.70)	0.264	0.93 (0.62–1.40)	0.731
Black/brown skin colour	0.98 (0.51–1.91)	0.956				
Former or current smoker	0.89 (0.37–2.15)	0.802				
Physical activity >150 min/week	0.88 (0.40–1.94)	0.756	1.09 (0.49–2.44)	0.839	1.06 (0.46–2.46)	0.891
Hazardous alcohol intake (AUDIT ≥8)	0.88 (0.40–1.93)	0.750	1.67 (0.66–4.19)	0.277	0.55 (0.22–1.38)	0.204
Metabolic features						
Obesity (BMI ≥30 kg/m^2^)	5.50 (2.85–10.6)	<0.001			4.28 (1.96–9.33)	<0.001
Type 2 diabetes	3.42 (1.73–6.78)	<0.001			1.56 (0.70–3.48)	0.280
Hypertension	4.84 (2.41–9.74)	<0.001			3.18 (1.30–7.76)	0.011
Dyslipidaemia	1.76 (0.84–3.69)	0.132			1.08 (0.47–2.48)	0.864
Weight gain ≥10% of body weight during follow‐up	0.75 (0.35–1.61)	0.459				
Liver disease and liver tests						
MASLD	4.58 (2.38–8.83)	<0.001	4.80 (2.20–10.5)	<0.001		
ALT level (per 10 U/L)	1.09 (0.94–1.26)	0.258				
AST level (per 10 U/L)	1.20 (0.94–1.53)	0.134				
HIV‐related factors						
CD4 count <350 cells/mm^3^	1.07 (0.41–2.75)	0.894	1.00 (0.39–2.59)	0.997	0.93 (0.31–2.76)	0.897
Detectable HIV‐RNA (>100 copies/mL)	0.49 (0.12–2.02)	0.322				
Use or switch to INSTI (vs no use during follow‐up)	0.88 (0.45–1.69)	0.691				
Duration of protease inhibitor use (per year)	0.99 (0.97–1.01)	0.329				
Duration of INSTI use (per year)	1.01 (0.96–1.07)	0.598				
Duration of PI use (per year)	0.99 (0.97–1.01)	0.329				
Duration of use of AZT, ddI, d4T or ddC (per year)	0.99 (0.94–1.05)	0.801				

*Note*: Multivariate models were adjusted for sex at birth, age, physical activity, hazardous alcohol intake and CD4 count. MASLD was entered in Multivariate Model A. Metabolic features replaced MASLD in Multivariate Model B to avoid collinearity. Physical activity was assessed in minutes of moderate‐intensity physical activity per week. Multivariate Model A: 292 participants and 35 outcomes. Multivariate Model B: 276 participants and 32 outcomes.

Abbreviations: aHR, adjusted hazard ratio; ALT, alanine transferase; AST, aspartate transferase; AUDIT, Alcohol Use Disorders Identification Test; AZT, zidovudine; BMI, body mass index; d4T, stavudine; ddC, zalcitabine; ddI, didanosine; HR, hazard ratio; INSTI, integrase strand transfer inhibitors; LSM, liver stiffness measurement; MASLD, metabolic dysfunction‐associated steatotic disease; PI, protease inhibitor.

Similar results were observed in a sensitivity analysis to identify risk factors associated with development of advanced fibrosis [*n* = 26; 8.6% (95% CI 5.9–12.3)], defined as LSM ≥9.5 kPa. In multivariate Cox Model A, MASLD [aHR = 3.43 (95% CI 1.36–8.62)] was independently associated with incident advanced fibrosis. When replacing MASLD by cardiometabolic risk factors (Cox Model B), type 2 diabetes [aHR = 2.91 (95% CI 1.07–7.92)] and hypertension [aHR = 5.20 (95% CI 1.59–16.94)] were associated with advanced fibrosis after adjustment for confounding factors (Table 5).

**TABLE 5:** Cox proportional hazards models to identify risk factors associated with incidence of advanced liver fibrosis (*n* = 26; LSM ≥9.5 kPa) in people living with HIV mono‐infection without liver fibrosis (LSM <8 kPa) at baseline followed up in the PROSPEC‐HIV cohort (*n* = 304) during a median time of 7.4 (IQR, 6.0–8.3) years.

	Univariate analysis		Multivariate Model A		Multivariate Model B	
	HR (95% CI)	*p* value	aHR (95% CI)	*p* value	aHR (95% CI)	*p* value
Socio‐demographic characteristics and lifestyle						
Male sex at birth	1.38 (0.64–2.99)	0.409	1.38 (0.62–3.07)	0.430	1.07 (0.41–2.76)	0.893
Age (per 10 years)	1.25 (0.87–1.80)	0.232	1.18 (0.80–1.75)	0.430	0.70 (0.43–1.14)	0.150
Black/brown skin colour	0.93 (0.42–2.04)	0.846				
Former or current smoker	0.57 (0.17–1.90)	0.358				
Physical activity >150 min/week	1.52 (0.65–3.52)	0.331	2.06 (0.85–5.00)	0.109	2.43 (0.95–6.19)	0.063
Hazardous alcohol intake (AUDIT ≥8)	0.80 (0.30–2.13)	0.654	1.27 (0.42–3.85)	0.677	0.38 (0.10–1.37)	0.139
Metabolic features						
Obesity (BMI ≥30 kg/m^2^)	3.42 (1.55–7.53)	0.002			1.81 (0.66–4.92)	0.246
Type 2 diabetes	3.87 (1.73–8.65)	0.001			2.91 (1.07–7.92)	0.036
Hypertension	5.18 (2.24–12.01)	<0.001			5.20 (1.59–16.94)	0.006
Dyslipidaemia	1.48 (0.63–3.47)	0.364			1.19 (0.43–3.25)	0.741
Weight gain ≥10% of body weight during follow‐up	0.70 (0.28–1.77)	0.447				
Liver disease and liver tests						
MASLD	3.50 (1.59–7.73)	0.002	3.43 (1.36–8.62)	0.009		
ALT level (per 10 U/L)	1.07 (0.88–1.29)	0.509				
AST level (per 10 U/L)	1.22 (0.91–1.64)	0.177				
HIV‐related factors						
CD4 count <350 cells/mm^3^	1.34 (0.46–3.93)	0.593	1.57 (0.52–4.77)	0.428	1.93 (0.52–7.16)	0.324
Detectable HIV‐RNA (>100 copies/mL)	0.75 (0.18–3.17)	0.693				
Use or switch to INSTI (vs no use during follow‐up)	0.88 (0.40–1.94)	0.755				
Duration of protease inhibitor use (per year)	0.98 (0.96–1.01)	0.231				
Duration of INSTI use (per year)	1.01 (0.95–1.08)	0.662				
Duration of PI use (per year)	0.98 (0.96–1.01)	0.231				
Duration of use of AZT, ddI, d4T or ddC (per year)	0.98 (0.92–1.05)	0.572				

*Note*: MASLD was entered in Multivariate Model A. Metabolic features replaced MASLD in Multivariate Model B to avoid collinearity. Physical activity was assessed in minutes of moderate‐intensity physical activity per week. Multivariate Model A: 292 participants and 25 outcomes. Multivariate Model B: 276 participants and 22 outcomes.

Abbreviations: aHR, adjusted hazard ratio; ALT, alanine transferase; AST, aspartate transferase; AUDIT, alcohol use disorders identification test; AZT, zidovudine; BMI, body mass index; c‐ART, combined antiretroviral therapy; d4T, stavudine; ddC, zalcitabine; ddI, didanosine; HR, hazard ratio; INSTI, integrase strand transfer inhibitors; MASLD, metabolic dysfunction‐associated steatotic liver disease; Met‐ALD, metabolic dysfunction‐ and alcohol‐associated liver disease; PI, protease inhibitor.

## DISCUSSION

This prospective longitudinal study highlights that the presence of MASLD increases the risk of developing CSF in people with HIV mono‐infection under c‐ART. Moreover, the present data reinforced that metabolic features, particularly BMI ≥30 kg/m^2^, hypertension and type‐2 diabetes, were independently associated with incident liver fibrosis in PLWH. The incidence of CSF in people with HIV infection would be more likely driven by components of the metabolic syndrome, such as obesity, rather than the presence of MASLD. On the other hand, the duration of c‐ART and the use of INSTI (yes vs. no and ≥12 months vs. <12 months) were not associated with incident liver fibrosis. The findings presented here underscore the importance of integrating strategies and early interventions to promote a healthy lifestyle and to prevent metabolic disorders, especially obesity, hypertension and type 2 diabetes, in PLWH.

One study has shown that the presence of advanced liver fibrosis/cirrhosis seems to be the landmark for increased risk of liver‐related complications and/or all‐cause mortality in people from the general population without HIV infection [19]. A retrospective cohort study of 646 individuals with biopsy‐proven NAFLD followed up for 20 years reported that the risk of liver‐specific morbidity increased per stage of fibrosis [13]. Similar results were observed in two multicentre prospective studies that followed large cohorts of people from the general population with biopsy‐confirmed NAFLD [20, 21]. The increased risk of overall mortality and liver‐related morbidity as per fibrosis stage in people with MASLD/NAFLD was reinforced by a systematic review/meta‐analysis that included 13 studies comprising 4428 subjects without HIV infection with MASLD [22].

Evidence of the burden of MASLD in PLWH has been described [8]. A systematic review that included 43 studies and reported data for 8230 patients described a pooled prevalence of moderate (F ≥ 2) and advanced liver fibrosis (F ≥ 3) in people with HIV mono‐infection of 12% (95% CI 10–14) and 5% (95% CI 2–8), respectively [23]. More recently, Gawrieh et al. have reported a prevalence of 15% for CSF (8 kPa) and 4% for cirrhosis (12 kPa) using TE in a multicentre US study that evaluated 1065 PLWH [10]. Additionally, a recent Spanish multicentre study including 2151 PLWH has described that the presence of advanced fibrosis measured by LSM increased the risk of overall death and liver‐related death in PLWH [24].

Although robust evidence‐based reporting the burden of MASLD in PLWH, data on the prognostic value of MASLD for liver fibrosis progression remain scarce. A Canadian prospective study reported a relatively low incidence of liver fibrosis progression (4% achieved advanced liver fibrosis defined by Fibrosis‐4 score (FIB‐4) ≥3.25, incidence = 9 per 1.000 PY) in 796 subjects with HIV mono‐infection followed up for a median of 4.9 (IQR, 2.2–6.4) years [25]. The present study reported a higher incidence rate [17.3 (95% CI 12.5–23.9) per 1.000 PY] of incident CSF. This might be explained by the use of different non‐invasive methods for defining liver fibrosis in both studies. Additionally, FIB‐4 seems to be better to exclude rather than to rule in the presence of advanced liver fibrosis [26]. Bischoff et al. showed that LSM and scores indicating liver disease progression [Fibroscan‐AST, Aspartate‐Platelet‐Ratio‐Index and FIB‐4 scores] increased significantly during a mean follow‐up of 42 months with the appearance of steatosis in 319 people with HIV mono‐infection [27]. Additionally, the presence of steatosis predicted liver fibrosis progression in HIV mono‐infection (aHR 4.18, 95% CI 1.21–14.5) in the LIVEHIV cohort (Canada) [28].

A more recent retrospective study has analysed data from 1183 participants from Canada, Italy and Germany followed up for 2.5 (1.9–3.5) years to describe incident liver fibrosis progression in PLWH [29]. The present study reported a slightly higher overall cumulative incidence (95% CI) of liver fibrosis [13% (2.3–18.0) vs. 8% (6.5–9.9)], probably related to the longer follow‐up. Similar to our results, MASLD was an independent predictor of liver fibrosis [aHR = 2.72 (95% CI 1.05–7.02)] in the multinational study. Additionally, neither HIV infection duration nor exposure to INSTI was associated with developing liver fibrosis in both studies. Contrary to our findings, they reported that weight gain (>5% on BMI) was independently associated with incident fibrosis [aHR = 3.12 (95% CI 1.41–6.90)]. Despite using a similar non‐invasive method (TE) and similar threshold to define liver fibrosis (8 kPa), there were few differences between both studies. In the retrospective multinational study, the primary outcome was liver fibrosis progression, defined as the development of significant liver fibrosis (LSM ≥8 kPa) for those with LSM <8 kPa at baseline or transition to cirrhosis (LSM ≥13 kPa) for those with LSM between 8 and 12.9 at baseline. Additionally, 25% of their participants had viral hepatitis co‐infection, and around 44% of PLWH were currently using INSTI at baseline. On the other hand, the present prospective study excluded people with CSF (LSM ≥8 kPa) at baseline or viral hepatitis co‐infection, focusing on people with HIV mono‐infection without liver fibrosis. Despite a relatively low proportion of participants under the INSTI regimen at baseline (7%), a total of 47% (*n* = 143) of our sample were exposed to INSTI at the last visit (users and switchers to INSTI). A recent brief report stated that changes in hepatic steatosis assessed by CAP after switching to INSTI‐based regimens do not seem to parallel weight gain [30].

This study has some limitations. PROSPEC‐HIV is a convenience sample of PLWH recruited in a single tertiary centre specialized in HIV care in Rio de Janeiro, Brazil. We acknowledge that the characteristics of our sample might be different from the current HIV epidemiology in Brazil where most of the new infections have been occurred in young adults. Therefore, our sample might not be representative of PLWH treated in Brazil. We have included mostly people aged >40 years with long‐term HIV infection and c‐ART use who would be at higher risk for NCDs. The lack of dual liver biopsy to assess liver fibrosis progression might be the major limitation of this study. Additionally, we recognize that the use of TE might have led to misclassification of CSF due to LSM variation, especially in borderline cases. However, TE remains an extensive validated non‐invasive method to assess liver fibrosis. Additionally, the LSM cut‐off applied in the study (LSM ≥8.0 kPa) is highly indicative of clinically relevant fibrosis and has been used in several studies that included general population and PLWH [31, 32]. We acknowledge that many participants were excluded due to the loss to follow‐up. This might be explained by the COVID‐19 pandemic, since Brazil was one of the countries highly impacted by this global crisis accounting for more than 700 000 COVID‐19 related deaths [33]. Additionally, FIOCRUZ was become a national reference centre playing a critical role in coordinating efforts to tackle the COVID‐19 pandemic since March 2020 [34]. The World Health Organization (WHO) declared that COVID‐19 was no longer a global health emergency on 5 May 2023 [35]. Therefore, the visits of the PROSPEC‐HIV cohort were suspended from 2020 to 2023. A total of 29% of participants (*n* = 89) had three visits, and 71% (*n* = 215) had two visits during follow‐up. Moreover, 16% (*n* = 48) were censored or developed the outcome in the second visit before the interruption due to the COVID‐19 pandemic. This transitory suspension of study visits might have impacted on our findings. Participants were not evaluated as 36‐weeks intervals as originally planned. Additionally, those participants who were excluded, mainly due to the absence of a second TE examination, had a higher proportion of people with type 2 diabetes than of those included in the study. On the other hand, these two groups have no significant differences according to age, sex at birth, liver tests and other cardiometabolic risk factors (Table S3). We are aware that unmeasured factors, such as unhealthy diet or insulin resistance (HOMA‐IR), might potentially have contributed to the development of CSF. Moreover, physical activity was assessed by ‘minutes per week’ rather than ‘metabolic equivalent of task’ (METs) and structured questionnaires to assess physical activity, such as global physical activity questionnaire or international physical activity questionnaire, were not used. Additionally, the multi‐society SLD consensus and clinical practice guidelines defined the predominance of Met‐ALD or ALD by weekly intake of alcohol in grams. In the present study, excessive alcohol intake to define ALD was determined by a high score using AUDIT >15, as recommended by a panel of experts on alcohol‐related liver disease [36]. Additionally, we adjusted multivariate models following the WHO recommendation to consider an individual physically active: at least 150 min of moderate‐intensity physical activity per week [37]. The lack of HIV‐negative controls might be a criticism of this study. There is a hypothesis that the prevalence of MASLD and/or CSF might be higher in PLWH than in controls [14, 38]. Nevertheless, the goal of this study was to evaluate the long‐term association of MASLD with the incidence of liver fibrosis in people with HIV mono‐infection from Brazil, rather than assessing HIV‐specific contribution to liver fibrosis progression. The prevalence of MASLD could be impacted by the chosen CAP cut‐off and by the recent recommendation for statin therapy for PLWH who are at low‐to‐intermediate risk of cardiovascular disease (REPRIEVE‐study) [39]. The relatively low proportion of people with MASLD (17.8%) might have limited power in subgroup analyses. We estimated that CAP ≥263 dB/m would be adapted to detect clinically significant steatosis, and by using this threshold, our findings would be comparable with other recent studies. CAP accuracy seems to be good, but optimal CAP cut‐offs might differ substantially across aetiologies [40]. People with steatosis using statin without other cardiometabolic risk factors would be classified as MASLD. A total of 85% of our participants (*n* = 257) were using statins to reduce LDL‐cholesterol at baseline. However, the PROSPEC‐HIV baseline visits occurred before the evidence of REPRIEVE‐study. Additionally, none of participants were classified as MASLD at baseline (*n* = 54) based exclusively on the use of lipid‐lowering medication.

This study has some strengths. Our longitudinal study was designed to describe the incidence of NCDs in PLWH, including liver fibrosis progression, through a long‐term follow‐up. Additionally, MASLD definition was consistent with current guidelines, and people with viral hepatitis co‐infection were excluded to reduce confounding. All procedures were performed on the same day; laboratory analysis was centralized, people were interviewed by trained investigators using a structured questionnaire, and TE examinations were performed after an overnight fasting by experienced operators following standard operating procedures.

In conclusion, this study showed that the presence of MASLD was a predictor of incident liver fibrosis in a large well‐characterized sample of people with HIV mono‐infection from Brazil. Metabolic features seem to be the major drivers of liver fibrosis progression in PLWH. However, further studies are needed to confirm whether the occurrence of liver fibrosis is independently driven by MASLD or whether other factors related to HIV might be involved. A better understanding of the natural history of fibrosis progression in PLWH, especially those with HIV mono‐infection and/or with MASLD, should lead to better therapeutic strategies, economic models and healthcare policy decisions.

## AUTHOR CONTRIBUTIONS

**Juliana Fittipaldi:** contributed to data collection, interpretation of data; drafting and critical revision of the manuscript. **Sandra W. Cardoso and Estevão Portela Nunes:** contributed to study concept and design, interpretation of data; critical revision of the manuscript. **Cristiane Fonseca De Almeida and Patricia Dias De Brito:** contributed to data collection, interpretation of data; critical revision of the manuscript. **Valdilea G. Veloso and Beatriz Grinsztejn:** contributed to study concept and design, study supervision, interpretation of data and critical revision of the manuscript. **Hugo Perazzo:** contributed to study concept and design, study supervision, data collection, statistical analysis, interpretation of data and drafting and critical revision of the manuscript.

## FUNDING INFORMATION

This study was supported by Fundação Carlos Chagas Filho de Amparo à Pesquisa do Estado do Rio de Janeiro (grant number E‐26/201.351/2021 to HP), and Conselho Nacional de Desenvolvimento Científico e Tecnológico (CNPq) (grant number 445957/2020‐4 to HP). The funders had no role in the study design, data collection and analysis, decision to publish or preparation of the manuscript.

## CONFLICT OF INTEREST STATEMENT

The authors have nothing to disclosure.

## ETHICS STATEMENT

The study protocol was approved by the Institutional Review Board (IRB) from INI/FIOCRUZ (IRB 32889514.4.0000.5262). All participants signed an informed consent form prior to enrolment in this study.

## Supporting information


**Data S1.** Supporting Information.

## Data Availability

The data that support the findings of this study are available from the corresponding author upon reasonable request.
