# Supplementary material for: Metabolic dysfunction‐associated liver disease predicts incident liver fibrosis in people with HIV mono‐infection: A cohort study
Source: HIV Med. 2025 Jul 23;26(10):1525–39. doi: 10.1111/hiv.70079 (PMC12497932; doi:10.1111/hiv.70079)
Supplement: Supplementary file 1 — Data S1. Supporting Information. [file HIV-26-1525-s001.docx]

**Metabolic dysfunction-associated liver disease predicts incident liver fibrosis in people with HIV mono-infection: a cohort study**

**Authors**

Juliana Fittipaldi ^1^; Sandra W Cardoso ^1^; Estevão Portela Nunes ^1^; Cristiane Fonseca de Almeida ^1^; Patricia Dias de Brito ^1^; Valdilea G Veloso ^1^, Beatriz Grinsztejn ^1^, Hugo Perazzo ^1^

**SUPPLEMENTARY MATERIAL**

|  | **Page** |
| --- | --- |
| **Data collected at every PROSPEC-HIV study visit** | 1 |
| **Supplementary Table 1.** Incidence rate [per 1,000 person-years (95% confidence interval)] of advanced fibrosis (LSM ≥ 12 kPa) in people living with HIV mono-infection without liver fibrosis (LSM < 8 kPa) at baseline followed in the PROSPEC-HIV cohort during a median time of 7.4 (IQR, 6.0-8.3) years | 2 |
| **Supplementary Table 2.** Cox proportional hazards models to identify risk factors associated with incidence of clinically significant fibrosis (n=19; LSM ≥ 8 kPa) in people living with HIV mono-infection without liver fibrosis (LSM < 8 kPa) and without MASLD at baseline followed in the PROSPEC-HIV cohort (n=250) during a median time of 7.4 (IQR, 6.0-8.3) years | 3 |
| **Supplementary Table 3**. Comparison of sociodemographic, clinical and laboratory characteristics at baseline of participants with HIV-mono infection included and not included in the study | 4 |

**Data collected at every PROSPEC-HIV study visit**

The following data were collected on the same day at every visit of the PROSPEC-HIV study:

- Anthropometric measures: weight (kg), height (m), waist circumference (cm), hip circumference (cm),

- Blood pressure (mmHg)

- Co-morbidities: type-2 diabetes (no vs yes), hypertension (no vs yes), dyslipidemia (no vs yes), HBV and/or HCV coinfection (no vs yes), other chronic diseases (open field)

- Co-medications: medication for glucose control (oral or injectable) (no vs yes), medication for blood pression treatment (no vs yes), statins (no vs yes), fibrates (no vs yes), current or previous viral hepatitis treatment (no vs yes), other medications (open field). For people with current or previous viral hepatitis treatment: medications names, month and year of start, month and year of end; for people with previous HCV treatment: sustained virological response vs failure

- Alcohol intake: AUDIT score (10 questions)

- Physical activity: number of activities per week, minutes of activity per session of moderate-intensity physical activity

- Smoking: never, former or current; for former and current smoking: year of start smoking; year of end of smoking; number of cigarettes per day

- Fasting blood samples: RBC and WBC, platelet count, fasting glucose (mg/dL), ALT (U/L), AST (U/L), GGT (U/L), alkaline phosphatase (U/L), total bilirubin (mg/dL), albumin (g/dL), total cholesterol (mg/dL), LDL-cholesterol (mg/dL), HDL-cholesterol (mg/dL) and triglycerides (mg/dL)

- Transient elastography by Fibroscan: operator, probe (M or XL), liver stiffness measurement (LSM in kPa), IQR of LSM (kPa) , IQR/LSM ratio (%), number of valid measures (n), number of invalid measures (n) , success rate: valid/total measures ratio (%), Controlled Attenuation Parameter (CAP in dB/m), IQR of CAP (dB/m)

**Supplementary Table 1**. Incidence rate [per 1,000 person-years (95% confidence interval)] of advanced fibrosis (LSM ≥ 9.5 kPa) in people living with HIV mono-infection without liver fibrosis (LSM < 8 kPa) at baseline followed in the PROSPEC-HIV cohort during a median time of 7.4 (IQR, 6.0-8.3) years

|  | **n** | **Number of outcomes** | **Incidence of CSF per 1,000 PY (95%CI)** | **Relative Risk (95%CI)** | **p value** |
| --- | --- | --- | --- | --- | --- |
| **Overall** | 304 | 26 | 12.3 (8.4-18.1) |  |  |
| **According to sex at birth** |  |  |  |  |  |
| Female | 172 | 13 | 10.8 (6.3-18.7) | Reference |  |
| Male | 132 | 13 | 14.3 (8.3-24.6) | 1.32 (0.61-2.85) | 0.477 |
| **According to Age** |  |  |  |  |  |
| Age < 50 yrs | 208 | 15 | 10.2 (6.2-17.0) | Reference |  |
| Age ≥ 50 yrs | 92 | 11 | 17.9 (9.9-32.3) | 1.75 (0.80-3.80) | 0.155 |
| **According to metabolic features** |  |  |  |  |  |
| Absence of obesity (BMI < 30 Kg/m2) | 246 | 14 | 8.2 (4.9-13.8) | Reference |  |
| Presence of obesity (BMI ≥ 30 Kg/m2) | 58 | 12 | 29.9 (17.0-52.7) | 3.65 (1.69-7.88) | < 0.001 |
| Absence of type 2 diabetes | 221 | 14 | 8.4 (5.0-14.2) | Reference |  |
| Presence of type 2 diabetes | 81 | 11 | 25.6 (14.2-46.3) | 3.06 (1.39-6.73) | 0.004 |
| Absence of hypertension | 196 | 8 | 5.8 (2.9-11.6) | Reference |  |
| Presence of hypertension | 102 | 18 | 25.8 (16.2-40.9) | 4.43 (1.93-10.18) | < 0.001 |
| Absence of dyslipidemia | 121 | 8 | 9.4 (4.7-18.7) | Reference |  |
| Presence of dyslipidemia | 174 | 16 | 13.4 (8.2-21.9) | 1.44 (0.62-3.36) | 0.400 |
| **According to ALT levels** |  |  |  |  |  |
| Normal ALT levels (< 40 U/L) | 222 | 15 | 9.8 (5.9-16.3) | Reference |  |
| Abnormal ALT levels (≥ 40 U/L) | 81 | 10 | 17.5 (9.4-32.5) | 1.78 (0.80-3.96) | 0.153 |
| **According to HIV control** |  |  |  |  |  |
| CD4 count ≥ 350 cells/mm3 | 256 | 21 | 11.8 (7.7-18.1) | Reference |  |
| CD4 count < 350 cells/mm3 | 40 | 4 | 14.3 (5.4-38.1) | 1.21 (0.42-3.53) | 0.725 |
| Undetectable HIV viral load | 269 | 24 | 12.8 (8.6-19.1) | Reference |  |
| Detectable HIV viral load | 34 | 2 | 8.8 (2.2-35.2) | 0.69 (0.16-2.91) | 0.607 |
| **According to use of INSTI** |  |  |  |  |  |
| No use of INSTI during follow-up | 161 | 15 | 13.6 (8.2-22.5) | Reference |  |
| Use or switch to INSTI during follow-up | 143 | 11 | 11.0 (6.1-19.8) | 0.81 (0.37-1.76) | 0.593 |
| Cumulative use of INSTI < 12 months | 164 | 14 | 12.5 (7.4-21.2) | Reference |  |
| Cumulative use of INSTI ≥ 12 months | 140 | 12 | 12.1 (6.9-21.3) | 0.96 (0.44-2.08) | 0.926 |
| **According to liver disease** |  |  |  |  |  |
| Absence of MASLD | 250 | 14 | 8.0 (4.8-13.6) | Reference |  |
| Presence of MASLD | 54 | 12 | 32.9 (18.7-57.9) | 4.10 (1.89-8.86) | < 0.001 |

Missing data for age (n=300 & outcomes=26); type-2 diabetes (n=302 & outcomes=25); hypertension (n=298 & outcomes=26); dyslipidemia (n=295 & outcomes=24); ALT levels (n=303 & outcomes=25); CD4 count (n=296 & outcomes=25); HIV viral load (n=303 & outcomes=26). INSTI, integrase strand transfer inhibitors; MASLD, metabolic dysfunction-associated steatotic liver disease; LSM, liver stiffness measurement

**Supplementary Table 2**. Cox proportional hazards models to identify risk factors associated with incidence of clinically significant fibrosis (n=19; LSM ≥ 8 kPa) in people living with HIV mono-infection without liver fibrosis (LSM < 8 kPa) and without MASLD at baseline followed in the PROSPEC-HIV cohort (n=250) during a median time of 7.4 (IQR, 6.0-8.3) years

|  | **Univariate analysis** | | **Multivariate Model A** | | **Multivariate Model B** | |
| --- | --- | --- | --- | --- | --- | --- |
|  | **HR (95% CI)** | **p value** | **aHR (95% CI)** | **p value** | **aHR (95% CI)** | **p value** |
| **Socio-demographic characteristics and lifestyle** | | | | | | |
| Male sex at birth | 1.29 (0.52-3.18) | 0.577 | 1.16 (0.57-2.33) | 0.685 | 1.14 (0.57-2.31) | 0.706 |
| Age (per 10 years) | 1.31 (0.87-1.98) | 0.191 | 0.96 (0.65-1.42) | 0.849 | 0.93 (0.63-1.38) | 0.723 |
| Black/Brown skin colour | 1.63 (0.59-4.52) | 0.350 |  |  |  |  |
| Former or current smoker | 0.93 (0.27-3.19) | 0.906 |  |  |  |  |
| Physical activity > 150 min/ week | 1.35 (0.51-3.54) | 0.548 | 0.90 (0.40-2.04) | 0.803 | 0.94 (0.42-2.14) | 0.887 |
| Hazardous alcohol intake (AUDIT ≥ 8) | 1.75 (0.70-4.36) | 0.228 |  |  | 0.76 (0.34-1.71) | 0.505 |
| **Metabolic features** | | | | | | |
| Obesity (BMI ≥ 30 Kg/m2) | **5.60 (2.24-14.0)** | **< 0.001** | **3.42 (1.61-7.27)** | **0.001** | **3.52 (1.67-7.42)** | **0.001** |
| Type-2 diabetes | **2.78 (0.97-7.90)** | **0.056** | 1.55 (0.70-3.41) | 0.282 | 1.55 (0.71-3.41) | 0.272 |
| Hypertension | **4.02 (1.60-10.11)** | **0.003** | **3.49 (1.49-8.16)** | **0.004** | **3.62 (1.53-8.58)** | **0.003** |
| Dyslipidemia | 1.47 (0.54-3.98) | 0.447 |  |  |  |  |
| Weight-gain ≥ 10% of body weight during follow-up | 0.59 (0.19-1.80) | 0.352 |  |  |  |  |
| **Liver disease and liver tests** | | | | | | |
| Met-ALD or ALD | **3.59 (1.02-12.55)** | **0.045** | 1.38 (0.40-4.77) | 0.510 |  |  |
| ALT level (per 10 U/L) | 1.04 (0.81-1.32) | 0.779 |  |  |  |  |
| AST level (per 10 U/L) | 1.16 (0.81-1.66) | 0.790 |  |  |  |  |
| **HIV-related factors** | | | | | | |
| CD4 count < 350 cells/mm^3^ | 0.76 (0.18-3.31) | 0.720 | 0.93 (0.31-2.78) | 0.903 | 0.93 (0.32-2.75) | 0.899 |
| Detectable HIV-RNA (> 100 copies/ml) | 0.42 (0.06-3.19) | 0.405 |  |  |  |  |
| Use or switch to INSTI (vs no use during follow-up) | 1.01 (0.41-2.50) | 0.975 |  |  |  |  |
| Duration of protease inhibitor use (per year) | 0.99 (0.92-1.08) | 0.908 |  |  |  |  |
| Duration of INSTI use (per year) | 1.02 (0.77-1.37) | 0.869 |  |  |  |  |
| Duration of PI use (per year) | 0.99 (0.97-1.02) | 0.667 |  |  |  |  |
| Duration of use of AZT, ddI, d4T or ddC (per year) | 1.01 (0.95-1.08) | 0.671 |  |  |  |  |

Multivariate models were adjusted for sex at birth, age, physical activity, hazardous alcohol intake and CD4 count. Presence of Met-ALD or ALD was entered in Multivariate Model A. Hazardous alcohol consumption (AUDIT ≥ 8) replaced Met-ALD/ALD in Multivariate Model B to avoid collinearity. ALD, alcohol-related liver disease; ALT, alanine transaminase; AST, aspartate transaminase; AUDIT, Alcohol Use Disorders Identification Test; AZT, zidovudine; BMI, body mass index; ddI, didanosine; ddC, zalcitabine; d4T, stavudine; INSTI, integrase strand transfer inhibitors; LSM, liver stiffness measurement; Met-ALD, metabolic dysfunction-associated steatotic liver disease (MASLD) with moderate (increased) alcohol consumption; PI, protease inhibitor. Physical activity was assessed in minutes of moderate-intensity physical activity per week.

**Supplementary Table 3**. Comparison of sociodemographic, clinical and laboratory characteristics at baseline of participants with HIV-mono infection included and not included in the study

|  | **People with HIV mono-infection not included**  **(n=345)** | **People with HIV mono-infection included**  **(n=304)** | **P value** |
| --- | --- | --- | --- |
| **Demographic characteristics** |  |  |  |
| Male sex at birth ^a^ | 153 (44.3) | 132 (43.4) | 0.81 |
| Age, years ^b^ | 45 (34-53) | 44 (36-52) | 0.68 |
| Black/Brown skin colour ^a^ | 173 (50.1) | 179 (58.9) | 0.028 |
| **Metabolic factors** |  |  |  |
| Obesity, BMI > 30 Kg/m^2 a^ | 84 (24.3) | 58 (19.1) | 0.11 |
| Type-2 diabetes ^a^ | 311 (90.1) | 81 (26.6) | < 0.001 |
| Hypertension ^a^ | 132 (38.3) | 102 (33.6) | 0.21 |
| Dyslipidemia ^a^ | 207 (60.0) | 174 (57.2) | 0.66 |
| **Lifestyle** |  |  |  |
| Physical activity > 150 min/week ^a^ | 70 (20.3) | 75 (24.7) | 0.18 |
| Smoker ^a^ | 73 (21.2) | 53 (17.4) | 0.23 |
| Hazard alcohol intake, AUDIT score ≥ 8 ^a^ | 86 (24.9) | 76 (25.0) | 0.98 |
| **Biochemistry** |  |  |  |
| ALT, UI/L ^b^ | 31 (23-46) | 29 (23-41) | 0.02 |
| AST, UI/L ^b^ | 25 (20-34) | 25 (20-33) | 0.51 |
| GGT, U/L ^b^ | 51 (34-83) | 44 (32-71) | 0.035 |
| Alkaline Phosphatase, U/L | 90 (72-112) | 89 (72-110) | 0.62 |
| Albumin, g/dL ^b^ | 3.9 (3.7-4.2) | 3.9 (3.7-4.2) | 0.360 |
| Fasting glucose, mg/dL ^b^ | 95 (89-103) | 92 (88-99) | 0.005 |
| Total cholesterol, mg/dL ^b^ | 182 (155-213) | 186 (160-219) | 0.25 |
| HDL-cholesterol, mg/dL ^b^ | 43 (34-54) | 43 (37-54) | 0.49 |
| LDL-cholesterol, mg/dL ^b^ | 109 (88-133) | 115 (92-140) | 0.092 |
| Triglycerides, mg/dL ^b^ | 123 (84-178) | 121 (84-168) | 0.62 |
| Platelet count, x10^9^/mm^3 b^ | 248 (207-290) | 250 (211-290) | 0.56 |
| **HIV infection and c-ART history** |  |  |  |
| Duration of HIV infection, years ^b^ | 9.0 (4.1-16.8) | 9.6 (5.3-15.6) | 0.20 |
| CD4 count < 350 cells/mm^3^ ^b^ | 663 (429-906) | 671 (471-871) | 0.63 |
| Undetectable HIV-RNA (< 100 copies/ml) ^a^ | 297 (86.1) | 269 (88.5) | 0.30 |
| Duration of c-ART, years ^b^ | 7.0 (3.2-14.4) | 7.1 (3.7-13.4) | 0.62 |
| Current use of PI | 147 (42.6) | 152 (50.0) | 0.12 |
| Current use of INSTI | 23 (6.7) | 22 (7.2) | 0.86 |
| **Liver disease** |  |  | 0.58 |
| No SLD | 240 (69.6) | 234 (77.0%) |  |
| MASLD | 64 (18.6) | 54 (17.8) |  |
| Met-ALD | 22 (6.4) | 16 (5.3) |  |

Data expressed as (a) absolute (%) or (b) median [IQR]. ALT, alanine transaminase; AST, aspartate transaminase; AUDIT, Alcohol Use Disorders Identification Test; BMI, body mass index; c-ART, combined antiretroviral therapy; CSF, clinically significant fibrosis (defined by LSM ≥ 8 kPa during follow-up); HDL, high-density lipoprotein; INSTI, integrase strand transfer inhibitors; LDL, low-density lipoprotein; MASLD, metabolic dysfunction-associated steatotic liver disease; Met-ALD, metabolic dysfunction- and alcohol- associated liver disease; PI, protease inhibitor; SLD, steatotic liver disease. MASLD was defined as presence of steatosis with at least one cardiometabolic risk factor without excessive alcohol intake. Physical activity was assessed in minutes of moderate-intensity physical activity per week.
